# A Single-Cell Culture System for Dissecting Microenvironmental Signaling in Development and Disease of Cartilage Tissue

**DOI:** 10.3389/fcell.2021.725854

**Published:** 2021-10-18

**Authors:** Jade Tassey, Arijita Sarkar, Ben Van Handel, Jinxiu Lu, Siyoung Lee, Denis Evseenko

**Affiliations:** ^1^Department of Orthopaedic Surgery, Keck School of Medicine of USC, University of Southern California, Los Angeles, CA, United States; ^2^Department of Stem Cell Research and Regenerative Medicine, University of Southern California, Los Angeles, CA, United States

**Keywords:** chondrogenic organoid, chondrosphere, single cell, clonogenicity, microenvironment, niche, arthritis

## Abstract

Cartilage tissue is comprised of extracellular matrix and chondrocytes, a cell type with very low cellular turnover in adults, providing limited capacity for regeneration. However, in development a significant number of chondrocytes actively proliferate and remodel the surrounding matrix. Uncoupling the microenvironmental influences that determine the balance between clonogenic potential and terminal differentiation of these cells is essential for the development of novel approaches for cartilage regeneration. Unfortunately, most of the existing methods are not applicable for the analysis of functional properties of chondrocytes at a single cell resolution. Here we demonstrate that a novel 3D culture method provides a long-term and permissive *in vitro* niche that selects for highly clonogenic, colony-forming chondrocytes which maintain cartilage-specific matrix production, thus recapitulating the *in vivo* niche. As a proof of concept, clonogenicity of *Sox9*^*IRES–E*GFP^ mouse chondrocytes is almost exclusively found in the highest GFP^+^ fraction known to be enriched for chondrocyte progenitor cells. Although clonogenic chondrocytes are very rare in adult cartilage, we have optimized this system to support large, single cell-derived chondrogenic organoids with complex zonal architecture and robust chondrogenic phenotype from adult pig and human articular chondrocytes. Moreover, we have demonstrated that growth trajectory and matrix biosynthesis in these organoids respond to a pro-inflammatory environment. This culture method offers a robust, defined and controllable system that can be further used to interrogate the effects of various microenvironmental signals on chondrocytes, providing a high throughput platform to assess genetic and environmental factors in development and disease.

## Introduction

Chondrocytes are specialized extracellular matrix-secreting cells that contribute to maintenance of healthy cartilage. Due to the avascular nature of cartilage, cellular maintenance and turnover is relatively low in adult and there is low regenerative potential upon injury or pathological microenvironments (Jayasuriya et al., 2016). The joint niche balances paracrine input from synovial and immune cells and thus regulates chondrocyte response in both homeostasis and in diseased states, as seen in aberrant microenvironmental situations such as arthritis where the niche can drive loss of healthy matrix, chondrocyte hypertrophy and formation of fibrocartilage (Sokolove and Lepus, 2013; Shkhyan et al., 2018; Roseti et al., 2019). Although immature chondrocytes have some proliferative capacity (Yasuhara et al., 2011; Li et al., 2013), in pathological conditions such as osteoarthritis, this potential is insufficient to overcome degenerative signals in the microenvironment. Pro-degenerative factors, particularly interleukin 1 (IL-1β) and IL-6 family cytokines such as oncostatin M (OSM), overwhelm and suppress this regenerative potential, inevitably leading to cartilage degradation (Ni et al., 2015; Shkhyan et al., 2018).

The role of SRY-box transcription factor 9 (SOX9) in both homeostatic and arthritic chondrocytes have been well described (Haag et al., 2008; Zhang et al., 2015). Because SOX9 expression has been best described as a regulator of chondrogenesis, chondrocyte proliferation, and a marker for immature chondrocytes (Leung et al., 2011), it has the potential to be used for further assessment of their regenerative response to microenvironmental signaling. Increases in SOX9 activity have been correlated with stimulation by growth factors such as insulin-like growth factor-I (IGF-I) and fibroblast growth factor-2 (FGF-2) in articular chondrocytes (Shi et al., 2015), signifying its role in responding to the paracrine signals present in the niche. Other growth factors such as leukemia inhibitory factor (LIF), transforming growth factor-beta 1 (TGF-β1) and low levels of bone morphogenetic protein-4 (BMP-4) have been shown by our group to inhibit excessive chondrocyte maturation and hypertrophy (Wu et al., 2013), further suggesting that supplementation of growth factors can preserve SOX9-expressing chondrocytes in an immature state.

To model development and disease, multiple *in vitro* methods have been used to culture chondrocytes, however, chondrocytes grown in a 2-dimensional (2D) monolayer dedifferentiate into a fibroblast-like cell, losing expression of extracellular matrix molecules such as glycosaminoglycans (GAGs), collagen 2, and aggrecan (Caron et al., 2012; Wu et al., 2014; Matak et al., 2017). This loss of chondrogenic phenotype severely hinders the use of 2D *in vitro* experiments to accurately represent *in vivo* biology. To prevent this dedifferentiation of chondrocytes, many 3D culture systems of varying composition that attempt to mimic the chondrogenic niche have been developed, including agarose (Buschmann et al., 1992), fibrin glue (Perka et al., 2000), alginate (Almqvist et al., 2001), synthetic hydrogels (Ko et al., 2016) or aggregation into a pellet (Caron et al., 2012). Derivation of chondrocytes from mesenchymal stromal cells (MSCs) in a high-density pellet culture has also been utilized to assess chondrogenic capacity (Ullah et al., 2012). While these 3D methods are superior to traditional 2D culture of chondrocytes, there are no methods that offer single-cell resolution, which would provide a critical asset to interrogate the genetic and microenvironmental factors that influence the proliferative capacity of chondrocytes.

Here we present a novel 3D *in vitro* culture system using methylcellulose (MC), a culture method most commonly used for hematopoietic progenitor cell growth and differentiation (Li et al., 2013), for long-term culture of single chondrocytes. By supplementing MC with media previously established for the maintenance of human embryonic stem cell-derived chondrocyte progenitors, termed Maintenance Media (MM; Ferguson et al., 2018), we select for clonogenic colony-forming chondrocytes from developing tissue or adult articular cartilage by offering a microenvironment that best recapitulates the natural progenitor niche. In this method, chondrocytes from mouse, pig, and human form chondrospheres that retain their chondrogenic phenotype and resemble native cartilage, providing a more accurate surrogate for *in vivo* studies. These chondrogenic organoids can be maintained for at least 8–10 weeks, showing long-term stability of structure, viability and chondrogenic phenotype in a novel *in vitro* setting. This model enables the delineation of the impacts of pro-inflammatory stimuli on chondrocytes in a controllable setting that mimics microenvironmental signaling observed in arthritis, offering a novel and permissive system to model arthritis in a dish.

## Materials and Methods

### Mouse Line and Breeding

Homozygous *Sox9*^IRES–EGFP^ (shortened to *Sox9*^GFP^ henceforth; Strain 030137, Chan et al., 2011) and corresponding wildtype C57BL/6J (Strain 000664) mice were purchased from Jackson Laboratories and used for all subsequent *in vitro* studies. All procedures and breeding involving mice were approved by the Institutional Animal Care and Use Committee of USC and was compliant with all relevant ethical regulations regarding animal research.

### Tissue Collection and Digestion

Mouse, human, and pig tissues were enzymatically digested for varying lengths of time at 37°C with mild agitation in digestion media consisting of DMEM/F12 (Corning) with 10% Fetal Bovine Serum (FBS; Corning), 1% penicillin/streptomycin/amphotericin B solution (P/S/A; Corning), 1 mg/mL dispase (Gibco), 1 mg/mL type 2 collagenase (Worthington), 10 μg/mL gentamycin (Teknova) and 100 μg/mL primocin (Invivogen). For mouse femoral heads, postnatal day 7 (P7) *Sox9*^GFP^ or C57BL/6J pups were sacrificed and both femoral heads were harvested. Each pup was harvested separately as a biological replicate. Femoral heads were lightly crushed with a mortar and pestle, then digested in digestion media for 4–6 h. For adult *Sox9*^GFP^ mouse knee joints, 4 months old or 1.5 years old knees were cut at the femur and the tibia and crushed lightly with a mortar and pestle. They were then placed in digestion media in an Erlenmeyer flask with a spin bar in 4°C overnight, then in 37°C for 4–6 h. For pig articular cartilage, 4–6 months old Yucatan minipig hind legs were purchased from Premier BioSource (formerly S&S Farms). The cartilage was shaved from the articular surface of the condyles, minced and digested in digestion media at 37°C for 16–24 h. For human samples, young adult human primary tissue samples aged 19–26 years and mature adult human primary tissue samples aged 57–60 years were obtained from National Disease Research Interchange (NDRI) and complied with current IRB approval. All donated material was anonymous, carried no personal identifiers, and was obtained after informed consent. Human cartilage was digested in the same manner as pig cartilage. All cells were washed with DPBS (Corning) after digestion and strained through a 70 μm filter (Fisher) before use.

### Methylcellulose and Liquid Culture Method of Chondrocytes

To culture isolated chondrocytes in a 3D environment, methylcellulose (H4100, StemCell Technologies) was resuspended in Maintenance Media (MM; Ferguson et al., 2018), which consists of DMEM/F12 (Corning) with 10% FBS (Corning) and 1% P/S/A (Corning) supplemented with fibroblast growth factor-2 (FGF2, 10 ng/mL), bone morphogenic protein-4 (BMP4, 1 ng/mL), insulin-like growth factor-1 (IGF1, 10 ng/mL), Leukemia Inhibitory Factor (LIF, 50 ng/L), Transforming Growth Factor-β1 (TGF-β1 10 ng/mL), and primocin (100 μg/mL). All supplemental growth factors were purchased from PeproTech Inc., and the final concentration of methylcellulose was 1%. Passage 0–1 chondrocytes were resuspended at low density (300–400 cells/mL) and plated in 6-well ultra-low attachment plates (Corning) for 3–4 weeks. *Sox9*^GFP^ femoral head cells were cultured in a hypoxic chamber with 5% O_2_, whereas human and pig chondrocytes were cultured in normoxia. To ensure moisture and nutrients remained available to the cells, 120–160 μL of liquid MM was distributed twice weekly on the surface of the wells. After 3–4 weeks, colonies were counted for clonogenicity. Inclusion criteria consisted of clones larger than ∼40 μm in diameter or greater than 5 cell divisions as observed under a compound light microscope. All images of clones in methylcellulose were taken on an Echo Revolve Inverted microscope and scale bars were added using FIJI software (ImageJ). For individual clone culture in liquid media, colonies were plucked with a wide-bore pipette tip, washed in 10% FBS + 1% P/S/A + DMEM/F12 media, and transferred into a 96-well U-bottom ultra-low attachment plate (Corning) containing 200 μL of liquid MM. For arthritis in a dish experiments, addition of Oncostatin M (OSM; 10 ng/mL, PeproTech Inc.) to Maintenance Media was used. The remaining colonies were harvested for quantitative Real-Time PCR (qPCR) by washing the well with DPBS and further dilution of the methylcellulose by subsequent washing and centrifugation with DPBS in a 50 mL conical vial. To maintain the individual spheres in liquid, 100–120 μL of media was removed and added as necessary.

### Flow Cytometry and FACS

All flow cytometry and FACS was performed on a BD FACSAria IIIu cell sorter. Mouse cells were washed twice in 1–2% FBS and stained with DAPI for viability. Populations of interest were sorted based on DAPI negativity, GFP expression, and lack of the following lineage markers: APC-Cy7: CD31 (endothelial cells), Ter119 (erythrocytes), and CD45 (leukocytes) at 1 μL antibody/10^6^ cells (BioLegend). Cells were directly sorted into DMEM/F12 containing 10% FBS with 1% P/S/A. Flow cytometry data was analyzed using FlowJo software.

### RNA Sequencing Library Preparation and Sequencing

Total RNA was isolated using QIAGEN RNeasy Mini kit and quantified using Qubit fluorometer (Thermo Fisher Scientific). Quality of the isolated RNA was checked using Agilent Bioanalyzer 2100. Universal Plus mRNA-Seq. Library with NuQuant (TECAN) was used to generate stranded RNA-seq libraries. Briefly, poly(A) RNA was selected followed by RNA fragmentation. Double stranded cDNA was generated thereafter using a mixture of random and oligo(dT) priming. The library was then constructed by end repairing the cDNA to generate blunt ends, ligation of Unique Dual Index (UDI) adaptors, strand selection and PCR amplification. Different adaptors were used for multiplexing samples in one lane. Quality of the library was checked using Agilent Bioanalyzer 2100. Sequencing was performed on Illumina HiSeq 3000 with single end 50 base pair reads.

### RNA Sequencing Data Analysis

Raw fastq files were analyzed in Partek flow (version 10.0.21.0801). Reads were aligned to mouse GRCm38 (mm10) genome using Gencode Release M25 reference using STAR aligner (version 2.7.3a) (Dobin et al., 2013). Transcript levels were quantified to the reference using Partek E/M with default parameters. Normalization was done using counts per million (CPM) method. Genes were considered to be differentially expressed based on fold change >5 and FDR < 0.01. Heatmap for normalized gene expression was generated using Morpheus software^1^. Gene ontology enrichment analysis for the differentially expressed genes was performed using DAVID (Huang et al., 2009).

### RNA Extraction and Quantitative Real-Time PCR

Total RNA was extracted using either the RNeasy Mini or Micro Kit (Qiagen) and cDNA was amplified using the Maxima First Strand cDNA Synthesis Kit (Thermo Fisher Scientific). Power SYBR Green (Applied Biosystems) RT-PCR amplification and detection was performed using an Applied Biosystems Step One Plus Real-Time PCR machine. The comparative Ct method for relative quantification (2-ΔΔCt) was used to quantitate gene expression, where results were normalized to ribosomal protein L7 (*RPL7*). Primer sequences used and corresponding GenBank Accession numbers are listed in Supplementary Table 1.

### Histology, Immunohistochemistry, and Immunofluorescence

Tissues were fixed in either 10% formalin or 4% paraformaldehyde and sectioned at 5 μm. For DAB immunohistochemical (IHC) staining on pig slides, sections were deparaffinized using standard procedures and antigen retrieval was performed by incubating the slides in 1X citrate buffer pH 6.0 (Diagnostic Biosystems) at 60°C for 30 min followed by 15 min cooling at room temperature. For IHC on mouse slides, the above procedure was used with the addition of Proteinase K (Sigma) digestion at 37°C for 5 min after citrate buffer antigen retrieval. Endogenous peroxidase activity was quenched by treating samples with 3% H_2_O_2_ for 10 min at room temperature (RT). Sections were then blocked in 2.5% normal horse serum for 20 min, then incubated with primary antibodies diluted in PBS with 0.1–1% BSA (Sigma) overnight at 4°C. Sections were washed three times with TBS +0.05% Tween 20 (TBST, Sigma) before addition of HRP-conjugated secondary antibody for 30–120 min incubation at RT. Sections were washed three times with TBST after secondary incubation and DAB substrate (Vector Laboratories) was added until positive signal was observed. Sections were then immediately washed with tap water, counterstained in hematoxylin for 1 min and washed again with tap water before dehydration and mounting. Secondary antibody only (no primary antibody) controls were used for IHC, and a list of all antibodies and their concentrations used can be found in Supplementary Table 2. Toluidine Blue staining was performed on deparaffinized sections in accordance with standard laboratory techniques. For immunofluorescent visualization of apoptosis and proliferation, the TdT-mediated dUTP nick-end labeling (TUNEL; Promega Corp.) assay and the EdU Click-iT Assay Kit (Thermo Fisher Scientific) was performed as described in the manufacturer’s protocols, respectively. For the EdU assay, 10 μM of EdU was added to liquid MM for 24 h prior to fixation. Fluorescent slides were counterstained with DAPI (10 μg/mL in DPBS) for 5 min. All slides were viewed using a ZEISS Axio Imager. A2 Microscope and images were taken using ZEN 2 software (Zeiss). Standard microscope camera settings were used and scale bars were added using FIJI software (ImageJ).

### Statistical Analysis

The number of biological replicates and types of statistical analyses for each experiment are indicated in the figure legends. All statistical analyses were performed using Prism (version 9.0, GraphPad Software Inc.). All results are presented as mean ± SEM where *p* < 0.05 was considered statistically significant.

## Results

### Murine *Sox9*^GFPHi^ Chondrocytes Are Clonogenic in a Novel 3D *in vitro* Culture System

**FIGURE 1:**
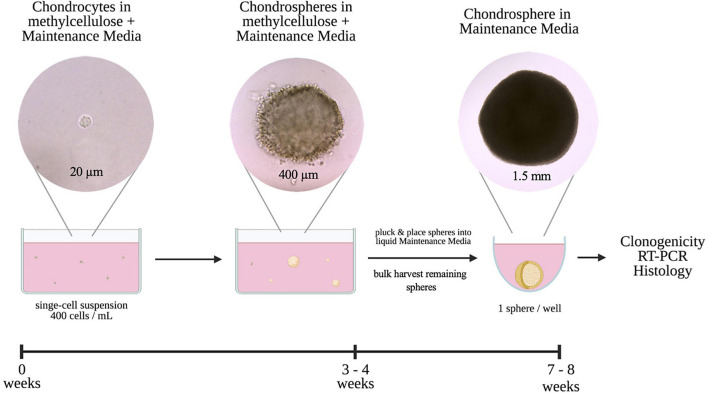
Schematic and timeline of 3D single-cell based *in vitro* culture method of colony-forming chondrocytes.

The main purpose of this study was to derive an *in vitro* method that recapitulated the 3D microenvironment to promote long-term retention of a chondrogenic phenotype at a single-cell level. To achieve this, chondrocytes were seeded at low density in methylcellulose (MC) reconstituted with Maintenance Media (MM) and cultured for 3–4 weeks (Figure 1). Once single cell-derived chondrogenic organoids, or chondrospheres, were established in MC, we transitioned the chondrospheres into liquid Maintenance Media to advance maturation for further assessment.

**FIGURE 2:**
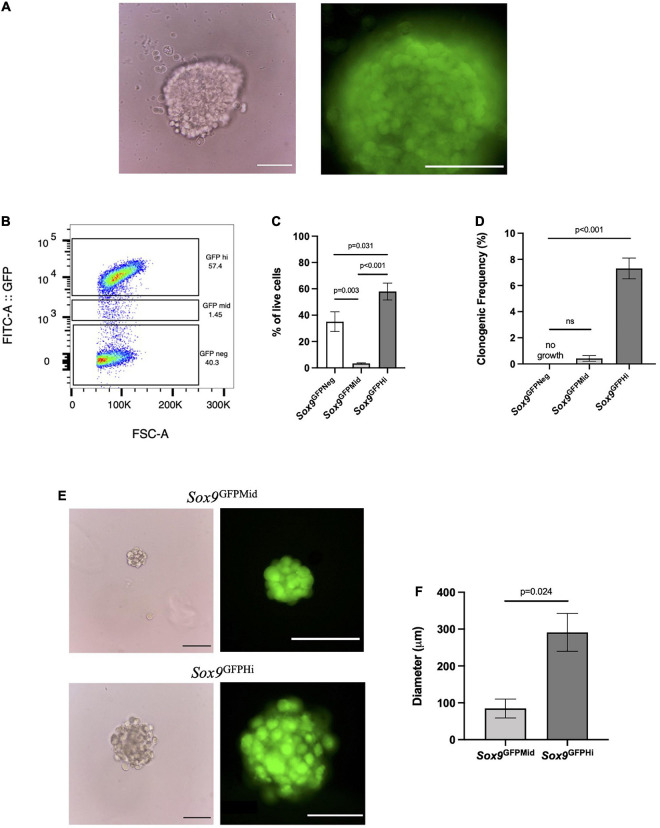
*Sox9*^GFPHi^ chondrocytes demonstrate increased clonogenic and proliferative capacity. **(A)** Unsorted P7 *Sox9*^GFP^ femoral chondrocytes after 3 weeks in methylcellulose (MC), Scale bar = 100 μm. **(B)** Representative FACS gating strategy and **(C)** frequency of live cells in *Sox9*^GFP^ fractions; *n* = 6 biological replicates and *p*-values calculated using one-way ANOVA with Tukey’s correction for multiple comparisons. **(D)** Clonogenicity of *Sox9*^GFP^ fractions in MC; *n* = 3 biological replicates and *p*-values calculated using One-Way ANOVA with Tukey’s correction for multiple comparisons. **(E)** Representative images of mid (upper row) and high (lower row) *Sox9*^GFP^ chondrospheres in MC after 4 weeks, scale bar = 100 μm. **(F)** Average diameter of chondrospheres in MC, *n* = 2 biological replicates, *p*-value calculated with unpaired *t*-test. All error bars represent mean ± SEM.

As a proof of concept, we wanted to address which cells contribute to colony formation by utilizing the *Sox9*^GFP^ reporter mouse (Figure 2). Due to an abundance of chondrocytes within the femoral heads of postnatal day 7 (P7) pups (Blumer et al., 2007), we isolated and digested the femoral heads and plated the cells in MC to see if *Sox9* expression was retained. These cells formed large chondrospheres that maintained high levels of GFP expression after 4 and 8 weeks in culture (Figure 2A and Supplementary Figure 1A, respectively). To interrogate if there were specific populations of *Sox9*-expressing cells generating the chondrogenic organoids, we fractionated cells via FACS based on level of *Sox9* expression into negative, mid or high GFP expression (Figure 2B) using wildtype P7 femoral head cells as a control (Supplementary Figure 1B). The negative and high fractions of *Sox9*^GFP^ cells represented about 40 and 57% the of the population, respectively, whereas the mid fraction represented less than 10% of live cells (Figure 2C), indicating an enrichment of *Sox9*^GFPHi^ cells endogenously found within the femoral heads. We plated the fractionated groups in MC, and after 4 weeks in MC, clonogenic frequency was assessed. A significant trend between increased clonogenicity and high *Sox9* expression was observed (Figure 2D), whereas cells in the *Sox9*^GFPNeg^ did not form any colonies. Furthermore, *Sox9* expression was detected in both the *Sox9*^GFPMid^ and *Sox9*^GFPHi^ fractions after culture in MC (Figure 2E), but the average diameter of the *Sox9*^GFPHi^ chondrospheres was about three-fold higher than the *Sox9*^GFPMid^ chondrospheres (Figure 2F), suggesting increased proliferative and/or matrix deposition capacity associated with *Sox9* expression, a trend also seen in the proliferating chondrocytes of the growth plate (Dy et al., 2012).

**FIGURE 3:**
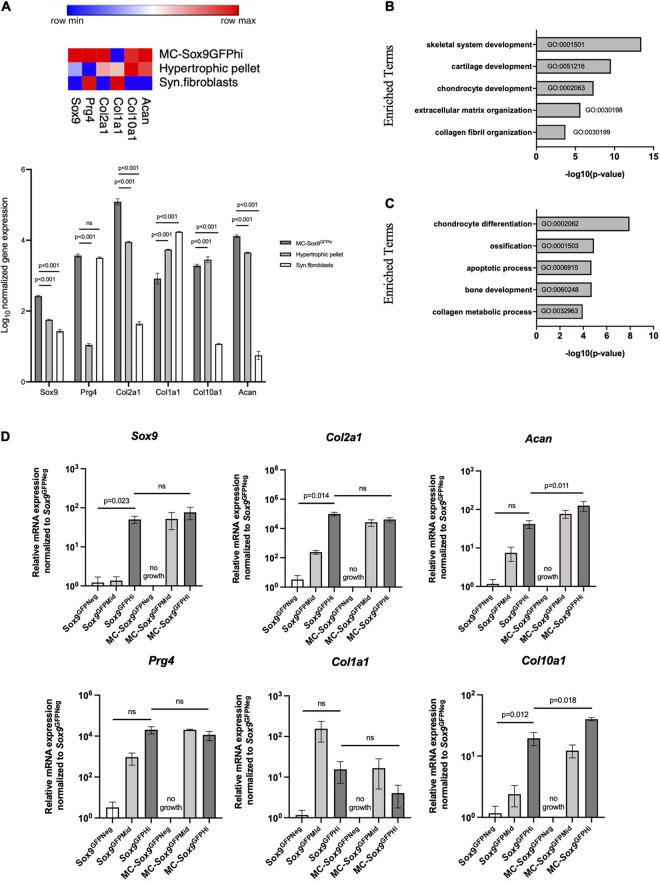
*Sox9*^GFP^ chondrocytes cultured in 3D culture maintain chondrogenic gene expression. **(A)** Heatmap and quantification showing the normalized bulk-sequencing gene expression profile for selected chondrogenic genes in *Sox9*^GFPHi^ chondrospheres, hypertrophic pellet, and synovial fibroblasts (Saeki and Imai, 2020 and Singh et al., 2021, respectively). Color scale denotes the minimum (blue) and maximum (red) expression value across each row. *P*-values were calculated with two-way ANOVA with Tukey’s correction for multiple comparisons. **(B)** Gene Ontology analysis showing enriched terms of *Sox9*^GFPHi^ chondrospheres versus synovial fibroblasts (*n* = 2 and 3, respectively). **(C)** Gene Ontology analysis showing enriched terms of hypertrophic pellets versus *Sox9*^GFPHi^ chondrospheres (*n* = 3 and 2, respectively). **(D)** Quantitative PCR analyses of chondrocyte-specific genes after normalizing expression to freshly isolated *Sox9*^GFPNeg^ chondrocytes; *n* = 4 biological replicates for freshly isolated *Sox9*^GFP^ fractions, *n* = 2 biological replicates for MC – *Sox9*^GFP^ fractions. *P*-values were calculated with one-way ANOVA with Tukey’s correction for multiple comparisons; all error bars represent mean ± SEM.

To better understand the differentiation pathways of the *Sox9*^GFPHi^ chondrospheres, transcriptomic analyses were performed. We used RNA-sequencing to compare the *Sox9*^GFPHi^ chondrospheres with previously published datasets using murine synovial fibroblasts to represent dedifferentiated chondrocytes commonly seen in long-term 2D culture (Saeki and Imai, 2020), and murine chondrocytes cultured in a 3D pellet assay designed for hypertrophic differentiation to represent terminally differentiated chondrocytes (Singh et al., 2021). The heat map for gene expression of selected chondrogenic genes indicated a clear enrichment in the *Sox9*^GFPHi^ chondrospheres after long-term *in vitro* culture when compared to fibroblasts or hypertrophic, pelleted chondrocytes (Figure 3A). Furthermore, Gene Ontology (GO) analyses of the genes significantly enriched (>5 fold-change, FDR < 0.01) in *Sox9*^GFPHi^ chondrospheres versus fibroblasts yielded categories related to cartilage and chondrocyte development, as well as extracellular matrix organization (Figure 3B). Conversely, when observing the genes significantly enriched in the hypertrophic pellet versus the *Sox9*^GFPHi^ chondrospheres, categories such as chondrocyte differentiation, ossification, and apoptotic process were upregulated (Figure 3C).

We then wanted to further validate the trends seen in the transcriptomic analyses via quantitative PCR performed on freshly isolated and cultured fractions of *Sox9*^GFP^ femoral chondrocytes (Figure 3D). Genes such as *Col2a1* and *Acan* in the freshly isolated *Sox9*^GFPHi^ fraction showed increased trends compared to freshly isolated *Sox9*^GFPNeg^ fraction and was maintained in the cultured *Sox9*^GFPMid^ and *Sox9*^GFPHi^ fractions. No cell growth was seen in the plated *Sox9*^GFPNeg^ fraction. *Col1a1* expression was relatively unchanged in the cultured chondrocytes, whereas *Col10a1* was enriched in both the freshly isolated and cultured *Sox9*^GFPHi^ cells (Figure 3D), suggesting that this culture system allows for natural differentiation of chondrocytes that are found in the femoral head. Of note, *Sox9* gene expression of freshly isolated *Sox9*^*GFPMid*^ chondrocytes was more akin to the *Sox9*^GFPNeg^ fraction, however, after 4 weeks in MC, cultured *Sox9*^GFPMid^ chondrocyte gene expression was more similar to the plated *Sox9*^GFPHi^ fraction, despite their differences in clonogenicity and size (Figures 2D,F).

**FIGURE 4:**
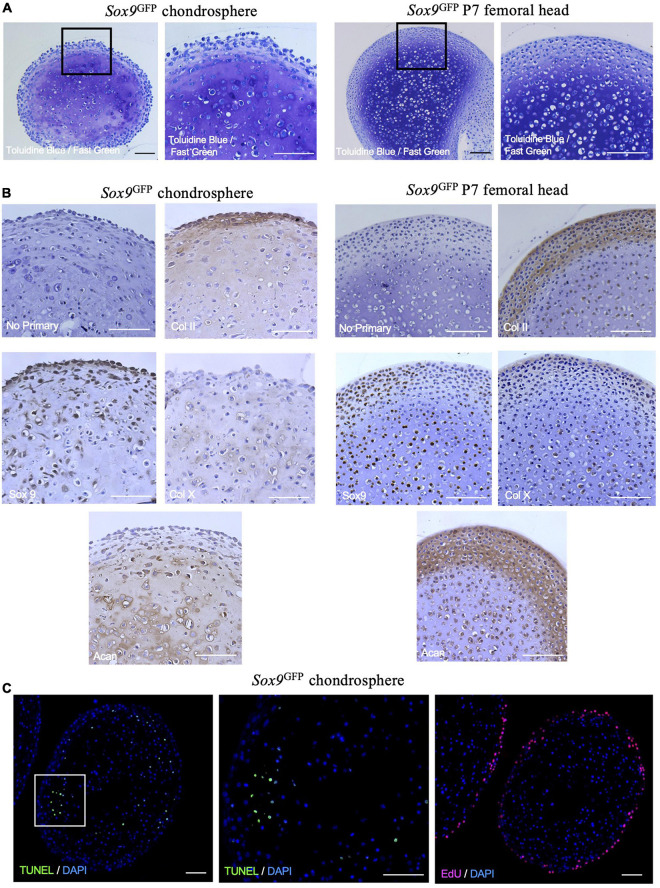
*Sox9*^GFP^ chondrospheres self-organize and form extracellular matrix similar to native femoral head after 8 weeks in culture. **(A)** Toluidine Blue/Fast Green staining for GAGs in *Sox9*^GFP^ chondrospheres (left) and P7 femoral head (right). **(B)** Immunohistochemistry of collagen II, Sox9, collagen X, and aggrecan in *Sox9*^GFP^ chondrospheres (left) and P7 femoral head (right). No primary antibody staining is provided as a negative control. **(C)** TUNEL staining to detect apoptosis (left, middle) and EdU staining to detect proliferation (right) in *Sox9*^GFP^ chondrospheres. Scale bar = 100 μm.

To assess the quality of matrix formed and organizational structure within the *in vitro* mouse chondrogenic organoids, we histologically compared *Sox9*^GFP^ chondrospheres grown for 8 weeks total to P7 femoral heads. Interestingly, the chondrospheres stained for GAGs comparably to femoral heads (Figure 4A) and collagen II was detected throughout the spheres (Figure 4B, upper right panels), but largely localized to the periphery as seen in the femoral head. Sox9 expression was also detected in the chondrospheres, as well as collagen X expression similar to the femoral head (Figure 4B, lower row). Both the mouse chondrospheres and the femoral head strongly stained for aggrecan (Figure 4B). When assessing apoptosis within the chondrospheres, TUNEL staining was sparse and randomly patterned, dissimilar to the centralized cell death observed in conventional aggregate pellet cultures (Figure 4C, left and middle; Weissenberger et al., 2020). Cell proliferation was observed at the periphery of the spheres (Figure 4C, right). Taken together, these results show that chondrocytes can form large and zonally structured multicellular organoids from a single cell *in vitro* and that the *Sox9*^GFPHi^ fraction exhibits the highest clonogenicity and chondrogenic capacity. Previously published literature states that colony-forming units in hematopoietic and mesenchymal systems are only typical for progenitor cells (Friedenstein et al., 1974; Sacchetti et al., 2007; Frisch and Calvi, 2014), suggesting that this system is preferentially selecting, based on their *in vivo* function, for the most immature chondrocytes with colony-initiating potential.

**FIGURE 5:**
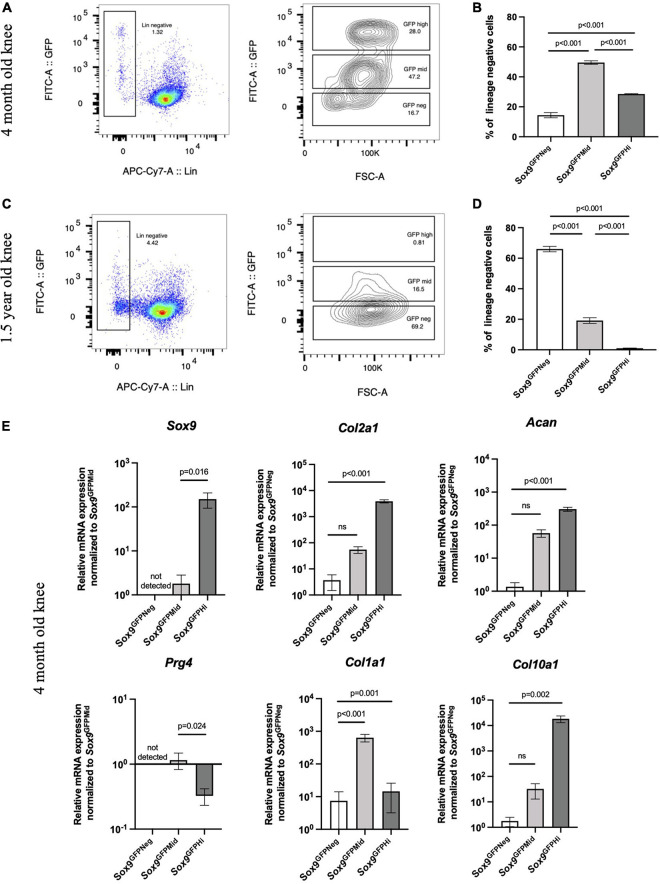
*Sox9*^GFPHi^ cells in the knee joint show highest chondrogenic gene expression but diminish with age. Representative FACS plot **(A)** and quantification **(B)** of *Sox9*^GFP^ levels in 4 months old *Sox9*^GFP^ knee joint; *n* = 3 biological replicates. Representative FACS plot **(C)** and quantification **(D)** of GFP levels in 1.5 years old *Sox9*^GFP^ knee joint; *n* = 3 biological replicates. **(E)** qPCR of chondrogenic genes in 4 months old *Sox9*^GFP^ cells in the knee joint, *n* = 6 biological replicates. Data represented as mean ± SEM and all *p*-values calculated using one-way ANOVA with Tukey’s correction for multiple comparisons.

Because proliferative capacity of chondrocytes declines with age, we assessed levels of *Sox9*^GFPHi^ cells in the mouse knee joints at different stage of ontogeny. We digested knee joints of young adult mice (4 months old) and mature adult mice (1.5 years old) and assessed GFP levels in the lineage negative fraction (CD45^–^/CD31^–^/Ter119^–^; Figure 5). Interestingly, *Sox9* expression shifted to favor the *Sox9*^GFPMid^ fraction in young adult mouse knee joints, and the *Sox9*^GFPHi^ fraction in knee joint was almost non-existent in mature adult mouse knee joints (Figures 5A–D). Furthermore, qPCR showed that the *Sox9*^GFPHi^ fraction isolated from young adult knees had the most chondrogenic gene expression except for *Prg4* (Figure 5E). When assessing gene expression in *Sox9*^GFPNeg^ and *Sox9*^GFPMid^ fractions of mature adult mouse knee joints, most chondrogenic genes were not detectable or not statistically significantly different from the *Sox9*^GFPNeg^ fraction (Supplementary Figure 1C). Altogether, this decrease in *Sox9* expression could indicate that a significant fraction of *Sox9*-expressing cells in the knee joint is lost with age.

### Human Articular Chondrocytes of All Ages Have Clonogenic Capacity

**FIGURE 6:**
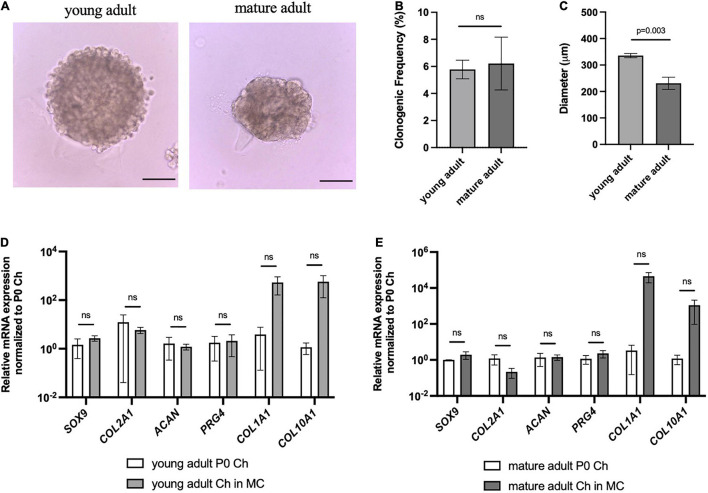
Human articular chondrocytes of different ages demonstrate clonality and retention of chondrogenic genes in 3D culture. **(A)** Representative images of young and mature adult chondrospheres in MC after 4 weeks, scale bar = 100 μm. **(B)** Clonogenic frequency of chondrocytes in MC, *n* = 2 biological replicates. **(C)** Diameter of chondrospheres of young and mature adult chondrospheres in MC, *n* = 2 biological replicates. qPCR of chondrogenic genes in young adult **(D)**, and mature adult **(E)** chondrospheres cultured in MC after normalizing expression to freshly isolated chondrocytes of respective samples, *n* = 2 biological replicates. Data represented as mean ± SEM and *p*-values calculated using an unpaired *t*-test.

Given that genetic reporters are not available for clinically relevant chondrocytes, we wanted to assess whether this *in vitro* culture system could be used to select for human colony-forming chondrocytes. Articular chondrocytes isolated from human young adult and mature adult were plated in our method (Figure 6). Intriguingly, we found that both age groups had cells capable of forming clones (Figure 6A). The young and mature adult chondrocytes had similar clonogenic frequency (Figure 6B), and for all tested donors only a very small percentage (around 5% or less) of articular chondrocytes formed multicellular organoids. Young adult chondrocytes formed significantly larger chondrospheres than mature adult chondrocytes (Figure 6C), and further analysis via qPCR showed that chondrogenic genes *SOX9*, *COL2A1*, and *ACAN* were maintained compared to freshly isolated cells (Figures 6D,E). However, while *COL1A1* and *COL10A1* showed upward trends in culture, these results were not statistically significant. These data indicate that while key chondrogenic genes are not lost, further optimization is needed to ensure that human chondrospheres do not transition into a fibrocartilage or hypertrophic phenotype.

### Porcine Articular Chondrocytes Maintain Chondrogenic and Clonogenic Capacity

**FIGURE 7:**
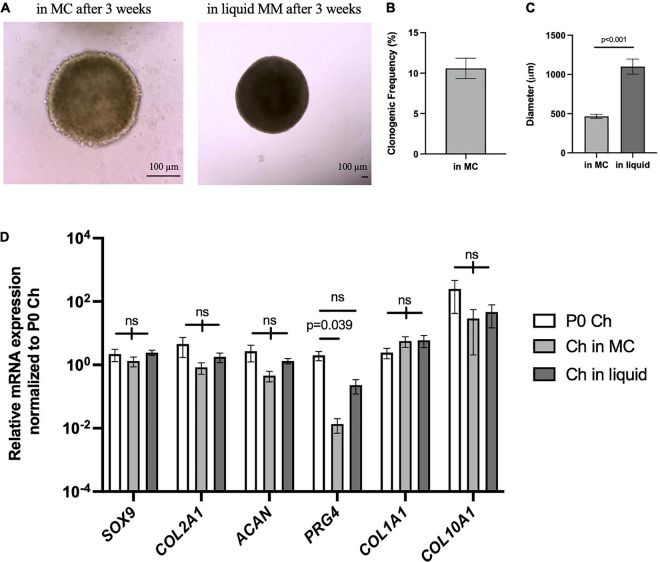
Porcine articular chondrocytes proliferate and maintain chondrogenic identity in 3D culture. **(A)** Representative images of pig chondrospheres in MC after 3 weeks (left) and after transition into liquid media for an additional 3 weeks (right); scale bar = 100 μm. **(B)** Clonogenic frequency of chondrospheres in MC, *n* = 9 biological replicates. **(C)** Average diameter of chondrospheres in MC versus liquid; *n* = 9 biological replicates, *p*-value calculated using an unpaired *t*-test. **(D)** qPCR of chondrogenic genes after normalizing expression to freshly isolated P0 chondrocytes; *n* = 9 biological replicates and *p*-value calculated by two-way ANOVA with Tukey’s correction for multiple comparisons. All data represented as mean ± SEM.

Next, we wanted to assess whether articular chondrocytes from a large animal used to model cartilage repair and arthritis can respond to the microenvironment of our 3D culture system. Chondrocytes isolated from porcine articular cartilage were cultured in MC for 3 weeks, where sizable chondrogenic organoids were formed (Figure 7A, left). Clonogenic frequency was similar to the *Sox9*^GFPHi^ fraction in mouse at 8–10% (Figure 7B). These porcine chondrospheres further grew in liquid Maintenance Media, reaching up to 1–1.5 mm in diameter (Figure 7A, right; Figure 7C), and compared to freshly isolated articular chondrocytes, chondrogenic gene expression was maintained even after long-term culture in our system (Figure 7D). *PRG4* expression decreased with culture in MC, however, began to increase back to native levels in the liquid phase of culture, suggesting reactivation of articular surface-specific markers.

**FIGURE 8:**
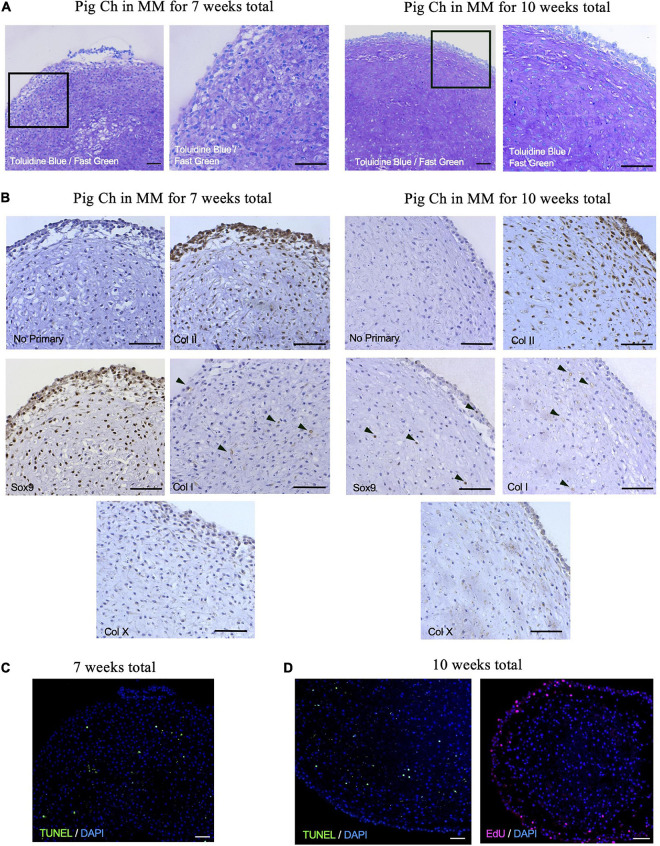
Porcine articular chondrospheres self-organize and deposit extracellular matrix. **(A)** Toluidine Blue/Fast Green staining for GAGs in pig articular chondrospheres cultured for 7 weeks (left) or 10 weeks total (right). **(B)** Immunohistochemistry of collagen II, Sox9, collagen I, and collagen X in pig articular chondrospheres cultured for 7 weeks (left) or 10 weeks total (right). Black arrows demarcate positive staining, no primary antibody staining is provided as a negative control. **(C)** TUNEL staining to detect apoptosis shown in chondrospheres cultured for 7 weeks. **(D)** TUNEL staining (left) and EdU staining to detect proliferation (right) shown in chondrospheres cultured for 10 weeks. Scale bar = 100 μm.

To further confirm that porcine articular chondrocytes retained their chondrogenic features after culture, we histologically assessed chondrospheres generated after 7- and 10-weeks (Figure 8). Similar to the P7 mouse chondrocytes, articular chondrocytes stained strongly for GAGs at both 7 and 10 weeks of culture (Figure 8A). Of note, immunohistochemical staining indicated that collagen II expression was present on the majority of chondrocytes, whereas SOX9 expression was enriched after 7 weeks but diminished at 10 weeks (Figure 8B), potentially reflecting the maturation process. Collagen I was also detected at minimal levels in pockets (Figure 8B), suggesting little to no fibrocartilage formation. TUNEL staining showed minimal and sparse pockets of apoptotic cells at both 7 and 10 weeks in culture (Figure 8C), suggesting retention of viability in the center of the sphere where hypoxia and lack of nutrients are often observed in larger aggregates (Ware et al., 2016). Proliferating chondrocytes at the periphery of the chondrogenic organoids were still detected after 10 weeks of culture in our system (Figure 8D). Altogether, these data reveal that this culture system can mimic a native niche to support clonogenic, colony-forming chondrocytes from a large animal, offering a customizable system that can be further used to interrogate pathophysiological environments.

### Arthritis in a Dish: Microenvironmental Cues Can Be Modified to Mimic Pro-degenerative Stimuli

**FIGURE 9:**
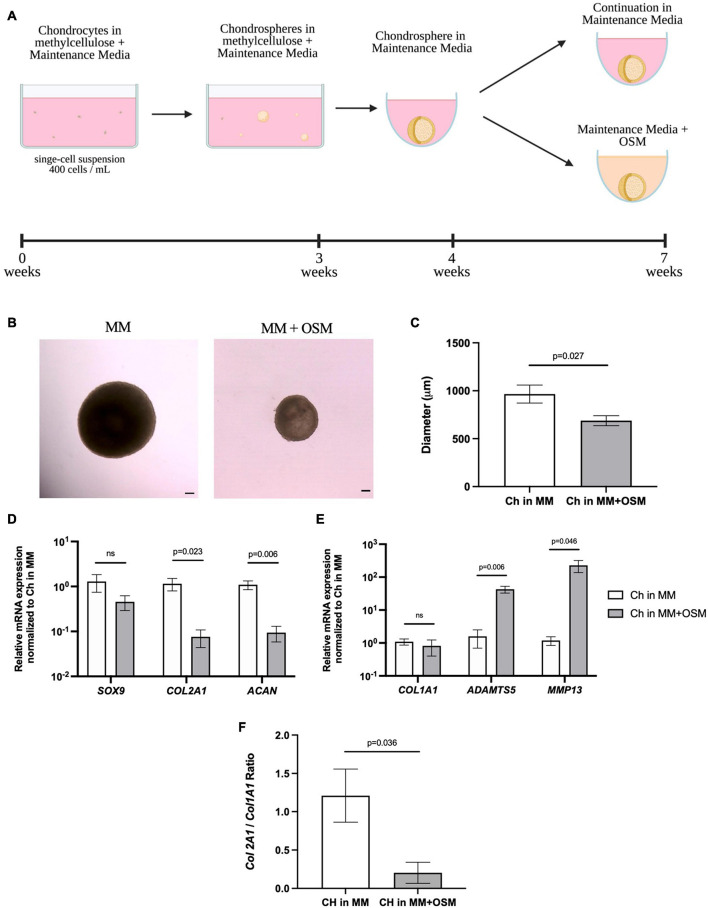
Porcine articular chondrospheres degrade and upregulate catabolic genes in response to pro-inflammatory stimuli. **(A)** Schematic and timeline of MC with MM culture method and introduction of pro-inflammatory cytokine OSM at 4 weeks. **(B)** Representative images of a pig articular chondrospheres in liquid MM alone (left) or liquid MM + OSM (right) after 3 weeks exposure, scale bar = 100 μm. **(C)** Average diameter of chondrospheres cultured in liquid MM or MM + OSM. **(D)** qPCR of anabolic genes after normalizing expression to chondrospheres cultured in MM alone, *n=4* biological replicates. **(E)** qPCR of catabolic genes after normalizing expression to chondrospheres cultured in MM alone, *n=4* biological replicates. **(F)** Collagen II/Collagen I ratio for chondrospheres in MM alone versus MM + OSM, *n=4* biological replicates. All data are represented as mean ±SEM and *p*-value calculated using an unpaired *t*-test.

After demonstrating that articular chondrocytes can form cartilage-like structures in our *in vitro* system, we then started manipulating microenvironmental factors to mimic a pro-inflammatory environment observed in arthritic joints. Porcine articular chondrocytes were cultured for 3 weeks in MC and transferred into liquid Maintenance Media for 1 week before the addition of the pro-inflammatory cytokine OSM for an additional 3 weeks (Figure 9A). Compared to chondrogenic organoids maintained in MM alone, chondrospheres exposed to pro-inflammatory cytokine OSM showed decreased density and size (Figures 9B,C). In response to OSM exposure, qPCR demonstrated that the spheres significantly downregulated chondrogenic genes *ACAN* and *COL2A1* but only marginally decreased *SOX9* expression (Figure 9D), an observation consistent with the early stages of osteoarthritic cartilage degeneration (Zhang et al., 2015). Although *COL1A1* was unchanged upon OSM treatment, significant *ADAMTS5* and *MMP13* upregulation indicated activation of catabolic pathways seen in degenerating cartilage (Figure 9E; Chan et al., 2017). The *COL2A1/COL1A1* ratio also showed a significant decrease in collagen II expression upon OSM exposure (Figure 9F), demonstrating a shift in the overall matrix composition. These results highlight the responsiveness of articular chondrocytes to pro-inflammatory stimuli most commonly found in diseased settings, showing a proof of concept for creating a dynamic and customizable *in vitro* readout for manipulating the joint niche. This will allow for assessment of genetic and microenvironmental factors that promote arthritic phenotypes in chondrocytes.

## Discussion

In this study, we have shown that chondrocytes can be cultured at a single-cell level in a 3D environment that recapitulates the *in vivo* niche, permitting them to retain their chondrogenic identity *in vitro*. We have demonstrated that three different species representing both small and large animals, as well as clinically relevant cells from human, show retention of the chondrogenic phenotype which allow for assessment of cells that can be genetically modified to interrogate molecular mechanisms of cellular regeneration and response to pro-inflammatory stimuli. While the use of methylcellulose culture has been described for assessing clonogenicity in hematopoietic cells (Matsui et al., 2004; Li et al., 2013), here it is used in conjunction with Maintenance Media, which provides chondrogenic factors and other factors preventing chondrocyte maturation (LIF) produced by synovial cells of both young and adult joints (Wu et al., 2013). Our *in vitro* model contributes to the maintenance of clonogenic colony-forming chondrocytes found in articular cartilage without promoting a fibrotic or hypertrophic phenotype, thus providing a more favorable physiological niche than traditional 2D culture systems allow. *In vivo* assays to assess feasibility and integration of chondrospheres into cartilage defects would be valuable and is our future direction, where improved production of large chondrospheres from patient autologous bio-specimens generated in this method has the potential to be therapeutically relevant for articular cartilage repair.

Based on the retention and selectivity of high *Sox9*^GFP^ expression in juvenile mouse femoral cells cultured in this method, it is likely that this culture system is only permissive for cells of chondrogenic lineage that are immature in nature. Deletion of *Sox9* in aggrecan-expressing cells inhibits chondrogenesis and chondrocyte proliferation (Akiyama et al., 2002; Dy et al., 2012), whereas a decrease in *Sox9* at different stages of development is essential for progression to a terminally differentiated, hypertrophic state (Leung et al., 2011). Because *Sox9* expression is retained even after 4 weeks in our culture system, it is feasible that this retention correlates to selection of an immature chondrocyte with minimal hypertrophy, our system recapitulating a progenitor niche, or a combination of both. Additionally, *Sox9*^GFPHi^ cells are minimally present in the articular cartilage of aged 1.5 years old mice. This finding is consistent with our previous studies in humans, showing that the percentage of SOX9 cells declines with age, and cells with high levels of SOX9 expression also express other progenitor markers such as GLI1, BMPR1B, and Integrin alpha 4, as well as activated STAT3 signaling (Ferguson et al., 2018).

Due to the natural development of the juvenile femoral head, there will be *Sox9*-expressing pre-hypertrophic cells, osteochondral precursor cells, and mesenchymal stromal cells that may co-express GAGs and collagen X where the secondary ossification center will form (Blumer et al., 2007; Madsen et al., 2011; Yen et al., 2021), which could explain the high gene expression levels of *Col10a1* seen by qPCR and RNA sequencing in both the freshly isolated and cultured *Sox9*^GFPHi^ fraction, respectively. Indeed, we observed the most differentiated, pre-hypertrophic/hypertrophic-like cells localized to the middle of the chondrogenic organoid. The immature and actively proliferating cells were found in the peripheral zone, concurrent with rare progenitor cells found on the superficial articular surface (Shwartz et al., 2016; Li et al., 2017), further suggesting self-organization of the cells *in vitro*. The increased expression levels of *Col1a1* within the *Sox9*^GFP^ mid fraction assessed by qPCR could also be due to a contamination of the *ligamentum teres femoris* when processing the femoral heads. Because this culture system is likely not permissive for cell types outside of the chondrocyte lineage, *Col1a1* expression in the *Sox9*^GFPMid^ or *Sox9*^GFPHi^ fraction is not maintained after culture. Overall, our data shows that *Sox9* expression correlates with the highest clonal and chondrogenic capacity and these cells are exclusively selected for in this system, allowing for future assessment of subpopulations found in immature clonogenic chondrocytes to advance our understanding of what drives regenerative potential.

Interestingly, human articular chondrocytes from young and mature adults demonstrate clonality in this culture system, but the percentage of cells with colony forming potential was relatively low in all tested specimens. Moreover, cultured cells showed an upward trend in *COL1A1* and *COL10A1* gene expression compared to freshly isolated cells, but neither the young or mature groups were statistically significant. Adult human cells did not show the same level of proliferation as the chondrocytes isolated from mice or pigs. Despite the multi-cellular appearance, no human chondrospheres of the age groups were large enough to move into the liquid phase of culture. This indicates the necessity of further optimization of culture conditions such as additional proliferation-inducing factors, or a hypoxic environment, which has also been shown to increase matrix accumulation specifically for human cell culture (Markway et al., 2013). Continual studies to further advance this culture technique for human cells are ongoing. Nevertheless, chondrogenic genes specific for hyaline-like cartilage were maintained, indicating some retention of a healthy chondrogenic phenotype.

It was interesting that porcine articular chondrocytes, considered a cell with limited self-renewal capacity, proliferated to such great extent that we observed. While it should be noted that porcine articular cartilage has been shown to spontaneously heal partial-thickness cartilage defects without bone marrow stimulation (Fisher et al., 2015), the level of growth observed in porcine articular chondrocytes was impressive. Diameters of up to 1–1.5 mm were reached with maintenance of chondrogenic genes expression, signifying highly anabolically active chondrocytes even after 6 weeks of total culture time. *COL10A1* expression was endogenously high in freshly isolated porcine articular chondrocytes, which have been previously described (Rucklidge et al., 1996), but could also be attributed to our chondrocyte harvest method including some hypertrophic chondrocytes found just above the subchondral bone. We did not detect hallmark fibrocartilage features as seen with minimal collagen I staining and *COL1A1* expression, supporting the superiority of our long-term culture method compared to 2D culture where cells rapidly de-differentiate and loose heir chondrogenic identity. Although there was a decrease in Sox9 detected via IHC by 10 weeks culture time, proliferative cells were still detected at the periphery of the chondrosphere as well as heavy GAG staining, validating our culture system’s ability to recreate a niche that supports progenitor cells long-term.

When looking at functional outcomes of porcine chondrospheres assessed in the arthritis in a dish model, exposure to the pro-inflammatory cytokine OSM significantly decreased the density and size of the spheres and upregulated catabolic genes most associated with ECM degradation and osteoarthritis (Heinegård and Saxne, 2011). This switch from anabolic to catabolic mechanisms of the chondrospheres due to OSM exposure supports previous data that show a decrease in aggrecan and TGF-β1 in chondrocytes cultured in 3D with OSM (Sanchez et al., 2004). This demonstrates the *in vitro* system’s capability of customization and multiplexing pro-inflammatory stimuli to assess potential anti-inflammatory or protective signaling in clinically relevant cells. Small molecule screening or cellular pathway inhibition are our future directions in further developing this readout for modulating outcomes of chondrospheres exposed to pro-inflammatory stimuli.

 Altogether, this novel *in vitro* culture system offers a robust and controllable system that can provide essential niche signaling to support and maintain colony forming chondrocytes at a single-cell level. Continual optimization will allow us to further perturbate responses of different chondrocyte subsets to extracellular stimuli in an *in vitro* model, which will be of great value for future genetic modifications to define genes required for regeneration in chondrocytes or development of new treatments specifically designed to enhances endogenous regenerative potential of articular cartilage tissue in diseases such as osteoarthritis.

## Data Availability Statement

The datasets presented in this study can be found in online repositories. The names of the repository/repositories and accession number(s) can be found below: https://www.ncbi.nlm.nih.gov/geo/, GSE184561.

## Ethics Statement

The animal study was reviewed and approved by the Institutional Animal Care and Use Committee of USC.

## Author Contributions

JT, BV, and DE conceptualized the study and interpreted the data. JT, AS, JL, and SL performed the experiments. JT wrote the manuscript. BV and DE revised and approved the manuscript. All authors contributed to the article and approved the submitted version.

## Conflict of Interest

The authors declare that the research was conducted in the absence of any commercial or financial relationships that could be construed as a potential conflict of interest.

## Publisher’s Note

All claims expressed in this article are solely those of the authors and do not necessarily represent those of their affiliated organizations, or those of the publisher, the editors and the reviewers. Any product that may be evaluated in this article, or claim that may be made by its manufacturer, is not guaranteed or endorsed by the publisher.

## Footnotes

1
https://software.broadinstitute.org/morpheus

## Supplementary Material

Supplementary Figure 1 Supplemental characterization of Sox9GFP chondrospheres, femoral head, and knee joints. **(A)** Representative FACS analysis of dissociated Sox9GFP spheres after 8 weeks total culture. **(B)** Representative FACS plot of GFP expression in wild-type P7 femoral head cells (left) and quantification (right) as percentage of live cells; *n=4* biological replicates, *p*-values calculated using one-way ANOVA with Tukey’s correction for multiple comparisons. **(C)** qPCR of chondrogenic genes in 1.5-year-old Sox9GFP cells in the knee joint, *n=3* biological replicates. Data are represented as mean ±SEM and *p*-values calculated using an unpaired *t*-test.

Supplementary Table 1 qPCR primer sequences.

Supplementary Table 2 Antibodies used for IHC.

## References

[B1] Akiyama H. Chaboissier M. C. Martin J. F. Schedl A. de Crombrugghe B. (2002). The transcription factor Sox9 has essential roles in successive steps of the chondrocyte differentiation pathway and is required for expression of Sox5 and Sox6. *Genes Dev.* 16 2813–2828. 10.1101/gad.1017802 12414734 PMC187468

[B2] Almqvist K. F. Wang L. Wang J. Baeten D. Cornelissen M. Verdonk R. (2001). Culture of chondrocytes in alginate surrounded by fibrin gel: characteristics of the cells over a period of eight weeks. *Ann. Rheum. Dis.* 60 781–790. 10.1136/ard.60.8.781 11454643 PMC1753804

[B3] Blumer M. J. Longato S. Schwarzer C. Fritsch H. (2007). Bone development in the femoral epiphysis of mice: the role of cartilage canals and the fate of resting chondrocytes. *Dev. Dyn.* 236 2077–2088. 10.1002/dvdy.21228 17626280

[B4] Buschmann M. D. Gluzband Y. A. Grodzinsky A. J. Kimura J. H. Hunziker E. B. (1992). Chondrocytes in agarose culture synthesize a mechanically functional extracellular matrix. *J. Orthop. Res.* 10 745–758. 10.1002/jor.1100100602 1403287

[B5] Caron M. M. Emans P. J. Coolsen M. M. Voss L. Surtel D. A. Cremers A. (2012). Redifferentiation of dedifferentiated human articular chondrocytes: comparison of 2D and 3D cultures. *Osteoarthritis Cartilage* 20 1170–1178. 10.1016/j.joca.2012.06.016 22796508

[B6] Chan C. M. Macdonald C. D. Litherland G. J. Wilkinson D. J. Skelton A. Europe-Finner G. N. (2017). Cytokine-induced MMP13 expression in human chondrocytes is dependent on activating transcription factor 3 (ATF3) regulation. *J. Biol. Chem.* 292 1625–1636. 10.1074/jbc.m116.756601 27956552 PMC5290940

[B7] Chan H. Y. Sivakamasundari S. Xing X. Kraus P. Yap S. P. Ng P. (2011). Comparison of IRES and F2A-based locus-specific multicistronic expression in stable mouse lines. *PLoS One* 6:e28885. 10.1371/journal.pone.0028885 22216134 PMC3244433

[B8] Dobin A. Davis C. A. Schlesinger F. Drenkow J. Zaleski C. Jha S. (2013). STAR: ultrafast universal RNA-seq aligner. *Bioinformatics* 29 15–21. 10.1093/bioinformatics/bts635 23104886 PMC3530905

[B9] Dy P. Wang W. Bhattaram P. Wang Q. Wang L. Ballock R. T. (2012). Sox9 directs hypertrophic maturation and blocks osteoblast differentiation of growth plate chondrocytes. *Dev. Cell* 22 597–609. 10.1016/j.devcel.2011.12.024 22421045 PMC3306603

[B10] Ferguson G. B. Van Handel B. Bay M. Fiziev P. Org T. Lee S. (2018). Mapping molecular landmarks of human skeletal ontogeny and pluripotent stem cell-derived articular chondrocytes. *Nat. Commun.* 9:3634. 10.1038/s41467-018-05573-y 30194383 PMC6128860

[B11] Fisher M. B. Belkin N. S. Milby A. H. Henning E. A. Bostrom M. Kim M. (2015). Cartilage repair and subchondral bone remodeling in response to focal lesions in a mini-pig model: implications for tissue engineering. *Tissue Eng. Part A* 21 850–860. 10.1089/ten.tea.2014.0384 25318414 PMC4333259

[B12] Friedenstein A. J. Deriglasova U. F. Kulagina N. N. Panasuk A. F. Rudakowa S. F. Luriá E. A. (1974). Precursors for fibroblasts in different populations of hematopoietic cells as detected by the in vitro colony assay method. *Exp. Hematol.* 2 83–92. 4455512

[B13] Frisch B. J. Calvi L. M. (2014). Hematopoietic stem cell cultures and assays. *Methods Mol. Biol.* 1130 315–324. 10.1007/978-1-62703-989-5_24 24482184 PMC4419375

[B14] Haag J. Gebhard P. M. Aigner T. (2008). SOX gene expression in human osteoarthritic cartilage. *Pathobiology* 75 195–199. 10.1159/000124980 18550917

[B15] Heinegård D. Saxne T. (2011). The role of the cartilage matrix in osteoarthritis. *Nat. Rev. Rheumatol.* 7 50–56. 10.1038/nrrheum.2010.198 21119607

[B16] Huang Dw Sherman B. T. Lempicki R. A. (2009). Systematic and integrative analysis of large gene lists using DAVID bioinformatics resources. *Nat. Protoc.* 4 44–57. 10.1038/nprot.2008.211 19131956

[B17] Jayasuriya C. T. Chen Y. Liu W. Chen Q. (2016). The influence of tissue microenvironment on stem cell-based cartilage repair. *Ann. N. Y. Acad. Sci.* 1383 21–33. 10.1111/nyas.13170 27464254 PMC5599120

[B18] Ko C. Y. Ku K. L. Yang S. R. Lin T. Y. Peng S. Peng Y. S. (2016). In vitro and in vivo co-culture of chondrocytes and bone marrow stem cells in photocrosslinked PCL-PEG-PCL hydrogels enhances cartilage formation. *J. Tissue Eng. Regen. Med.* 10 E485–E496. 10.1002/term.1846 24668937

[B19] Leung V. Y. Gao B. Leung K. K. Melhado I. G. Wynn S. L. Au T. Y. (2011). SOX9 governs differentiation stage-specific gene expression in growth plate chondrocytes via direct concomitant transactivation and repression. *PLoS Genet.* 7:e1002356. 10.1371/journal.pgen.1002356 22072985 PMC3207907

[B20] Li L. Newton P. T. Bouderlique T. Sejnohova M. Zikmund T. Kozhemyakina E. (2017). Superficial cells are self-renewing chondrocyte progenitors, which form the articular cartilage in juvenile mice. *FASEB J.* 31 1067–1084. 10.1096/fj.201600918r 27965322 PMC5295727

[B21] Li Q. Bohin N. Wen T. Ng V. Magee J. Chen S. C. (2013). Oncogenic Nras has bimodal effects on stem cells that sustainably increase competitiveness. *Nature* 504 143–147. 10.1038/nature12830 24284627 PMC4128640

[B22] Madsen S. H. Goettrup A. S. Thomsen G. Christensen S. T. Schultz N. Henriksen K. (2011). Characterization of an ex vivo femoral head model assessed by markers of bone and cartilage turnover. *Cartilage* 2 265–278. 10.1177/1947603510383855 26069585 PMC4300811

[B23] Markway B. D. Cho H. Johnstone B. (2013). Hypoxia promotes redifferentiation and suppresses markers of hypertrophy and degeneration in both healthy and osteoarthritic chondrocytes. *Arthritis Res. Ther.* 15:R92. 10.1186/ar4272 23965235 PMC3979022

[B24] Matak D. Brodaczewska K. K. Lipiec M. Szymanski Ł Szczylik C. Czarnecka A. M. (2017). Colony, hanging drop, and methylcellulose three dimensional hypoxic growth optimization of renal cell carcinoma cell lines. *Cytotechnology* 69 565–578. 10.1007/s10616-016-0063-2 28321776 PMC5507837

[B25] Matsui W. Huff C. A. Wang Q. Malehorn M. T. Barber J. Tanhehco Y. (2004). Characterization of clonogenic multiple myeloma cells. *Blood* 103 2332–2336. 10.1182/blood-2003-09-3064 14630803 PMC3311914

[B26] Ni J. Yuan X. M. Yao Q. Peng L. B. (2015). OSM is overexpressed in knee osteoarthritis and Notch signaling is involved in the effects of OSM on MC3T3-E1 cell proliferation and differentiation. *Int. J. Mol. Med.* 35 1755–1760. 10.3892/ijmm.2015.2168 25845347

[B27] Perka C. Spitzer R. S. Lindenhayn K. Sittinger M. Schultz O. (2000). Matrix-mixed culture: new methodology for chondrocyte culture and preparation of cartilage transplants. *J. Biomed. Mater. Res.* 49 305–311. 10.1002/(SICI)1097-4636(20000305)49:3<305::AID-JBM2>3.0.CO;2-9 10602062

[B28] Roseti L. Desando G. Cavallo C. Petretta M. Grigolo B. (2019). Articular cartilage regeneration in osteoarthritis. *Cells* 8:1305. 10.3390/cells8111305 31652798 PMC6912428

[B29] Rucklidge G. J. Milne G. Robins S. P. (1996). Collagen type X: a component of the surface of normal human, pig, and rat articular cartilage. *Biochem. Biophys. Res. Commun.* 224 297–302. 10.1006/bbrc.1996.1024 8702386

[B30] Sacchetti B. Funari A. Michienzi S. Di Cesare S. Piersanti S. Saggio I. (2007). Self-renewing osteoprogenitors in bone marrow sinusoids can organize a hematopoietic microenvironment. *Cell* 131 324–336. 10.1016/j.cell.2007.08.025 17956733

[B31] Saeki N. Imai Y. (2020). Reprogramming of synovial macrophage metabolism by synovial fibroblasts under inflammatory conditions. *Cell Commun. Signal.* 18:188. 10.1186/s12964-020-00678-8 33256735 PMC7708128

[B32] Sanchez C. Deberg M. A. Burton S. Devel P. Reginster J. Y. Henrotin Y. E. (2004). Differential regulation of chondrocyte metabolism by oncostatin M and interleukin-6. *Osteoarthritis Cartilage* 12 801–810. 10.1016/j.joca.2004.06.011 15450530

[B33] Shi S. Wang C. Acton A. J. Eckert G. J. Trippel S. B. (2015). Role of sox9 in growth factor regulation of articular chondrocytes. *J. Cell. Biochem.* 116 1391–1400. 10.1002/jcb.25099 25708223 PMC5210171

[B34] Shkhyan R. Van Handel B. Bogdanov J. Lee S. Yu Y. Scheinberg M. (2018). Drug-induced modulation of gp130 signalling prevents articular cartilage degeneration and promotes repair. *Ann. Rheum. Dis.* 77 760–769. 10.1136/annrheumdis-2017-212037 29436471 PMC8444286

[B35] Shwartz Y. Viukov S. Krief S. Zelzer E. (2016). Joint development involves a continuous influx of Gdf5-positive cells. *Cell Rep.* 15 2577–2587. 10.1016/j.celrep.2016.05.055 27292641 PMC4920976

[B36] Singh P. Lessard S. G. Mukherjee P. Rourke B. Otero M. (2021). Changes in DNA methylation accompany changes in gene expression during chondrocyte hypertrophic differentiation in vitro. *Ann. N. Y. Acad. Sci.* 1490 42–56. 10.1111/nyas.14494 32978775 PMC7990741

[B37] Sokolove J. Lepus C. M. (2013). Role of inflammation in the pathogenesis of osteoarthritis: latest findings and interpretations. *Ther. Adv. Musculoskelet. Dis.* 5 77–94. 10.1177/1759720X12467868 23641259 PMC3638313

[B38] Ullah M. Hamouda H. Stich S. Sittinger M. Ringe J. (2012). A reliable protocol for the isolation of viable, chondrogenically differentiated human mesenchymal stem cells from high-density pellet cultures. *Biores. Open Access* 1 297–305. 10.1089/biores.2012.0279 23514965 PMC3559221

[B39] Ware M. J. Colbert K. Keshishian V. Ho J. Corr S. J. Curley S. A. (2016). Generation of homogenous three-dimensional pancreatic cancer cell spheroids using an improved hanging drop technique. *Tissue Eng. Part C Methods* 22 312–321. 10.1089/ten.tec.2015.0280 26830354 PMC4827286

[B40] Weissenberger M. Weissenberger M. H. Gilbert F. Groll J. Evans C. H. Steinert A. F. (2020). Reduced hypertrophy in vitro after chondrogenic differentiation of adult human mesenchymal stem cells following adenoviral SOX9 gene delivery. *BMC Musculoskelet. Disord.* 21:109. 10.1186/s12891-020-3137-4 32066427 PMC7026978

[B41] Wu L. Bluguermann C. Kyupelyan L. Latour B. Gonzalez S. Shah S. (2013). Human developmental chondrogenesis as a basis for engineering chondrocytes from pluripotent stem cells. *Stem Cell Reports* 1 575–589. 10.1016/j.stemcr.2013.10.012 24371811 PMC3871393

[B42] Wu L. Gonzalez S. Shah S. Kyupelyan L. Petrigliano F. A. McAllister D. R. (2014). Extracellular matrix domain formation as an indicator of chondrocyte dedifferentiation and hypertrophy. *Tissue Eng. Part C Methods* 20 160–168. 10.1089/ten.tec.2013.0056 23758619 PMC3910562

[B43] Yasuhara R. Ohta Y. Yuasa T. Kondo N. Hoang T. Addya S. (2011). Roles of β-catenin signaling in phenotypic expression and proliferation of articular cartilage superficial zone cells. *Lab. Invest.* 91 1739–1752. 10.1038/labinvest.2011.144 21968810 PMC3759358

[B44] Yen Y. Chien M. Wu P. Hung S. (2021). PP2A in LepR+ mesenchymal stem cells contributes to embryonic and postnatal endochondral ossification through Runx2 dephosphorylation. *Commun. Biol.* 4:658. 10.1038/s42003-021-02175-1 34079065 PMC8172534

[B45] Zhang Q. Ji Q. Wang X. Kang L. Fu Y. Yin Y. (2015). SOX9 is a regulator of ADAMTSs-induced cartilage degeneration at the early stage of human osteoarthritis. *Osteoarthritis Cartilage* 23 2259–2268. 10.1016/j.joca.2015.06.014 26162802

