# Supplementary figures and images for: A Single-Cell Culture System for Dissecting Microenvironmental Signaling in Development and Disease of Cartilage Tissue
Source: Front Cell Dev Biol. 2021 Oct 18;9:725854. doi: 10.3389/fcell.2021.725854 (PMC8558457; doi:10.3389/fcell.2021.725854)

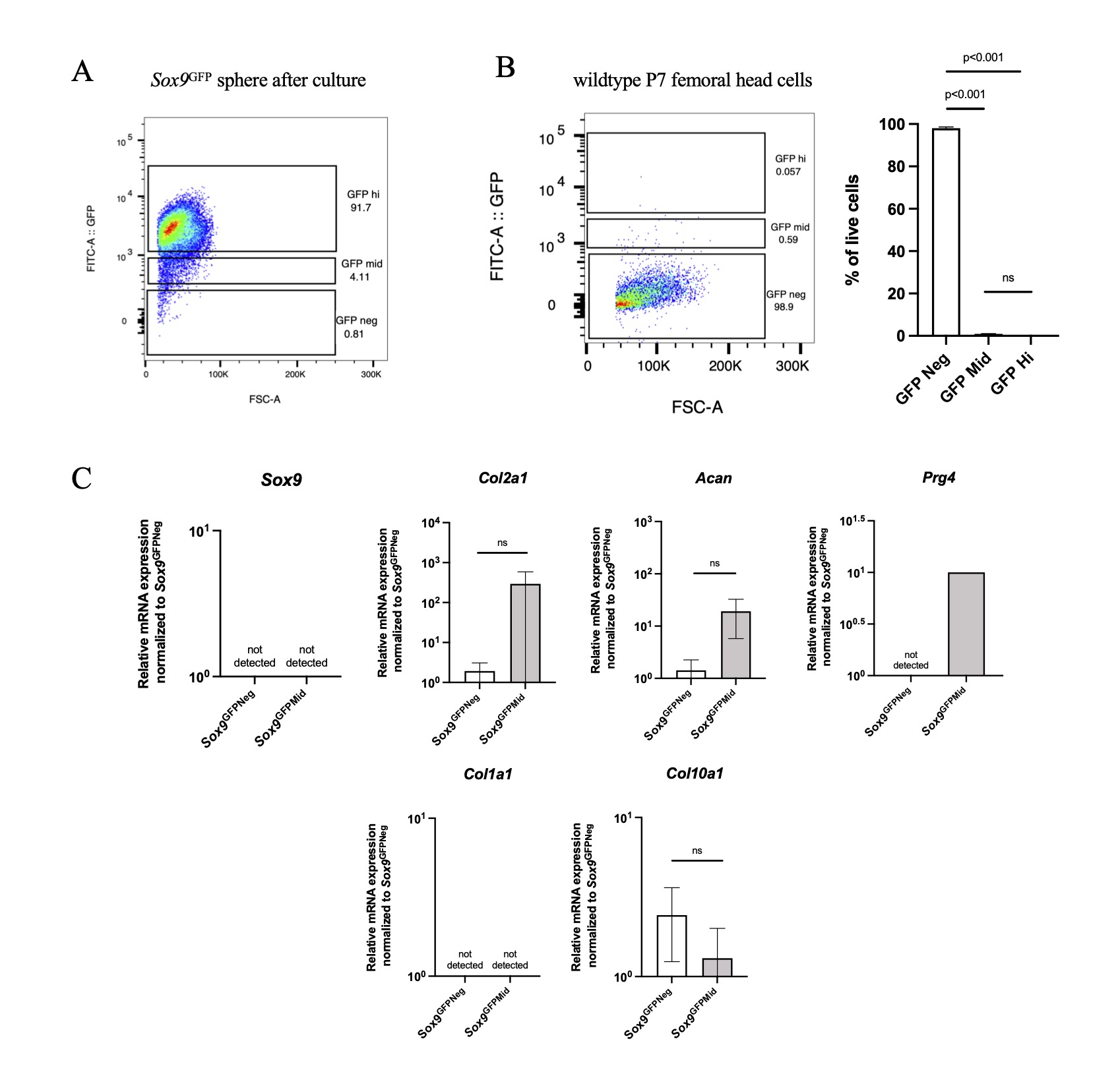

Supplement: Supplementary Figure 1 — Supplemental characterization of Sox9GFP chondrospheres, femoral head, and knee joints. (A) Representative FACS analysis of dissociated Sox9GFP spheres after 8 weeks total culture. (B) Representative FACS plot of GFP expression in wild-type P7 femoral head cells (left) and quantification (right) as percentage of live cells; n=4 biological replicates, p-values calculated using one-way ANOVA with Tukey’s correction for multiple comparisons. (C) qPCR of chondrogenic genes in 1.5-year-old Sox9GFP cells in the knee joint, n=3 biological replicates. Data are represented as mean ±SEM and p-values calculated using an unpaired t-test. [file Image_1.JPEG]
