# Supplementary material for: A Single-Cell Culture System for Dissecting Microenvironmental Signaling in Development and Disease of Cartilage Tissue
Source: Front Cell Dev Biol. 2021 Oct 18;9:725854. doi: 10.3389/fcell.2021.725854 (PMC8558457; doi:10.3389/fcell.2021.725854)
Supplement: Supplementary Table 1 — qPCR primer sequences. [file Table_1.DOCX]

**Supplemental Materials**

**Table S1 – qPCR primer sequences**

| **Gene name** | **Primer Sequence** | **GenBank Accession** |
| --- | --- | --- |
| mRPL7 | Forward: 5’ ACCGCACTGAGATTCGGATG 3’  Reverse: 5’ GAACCTTACGAACCTTTGGGC 3’ | NM_011291.5 |
| mSOX9 | Forward: 5’ CTGTACCTCCCTGAATAC 3’  Reverse: 5’ GCCAACAATCTACCTTAAA 3’ | NM_011448.4 |
| mCOL2A1 | Forward: 5’ GGGTCACAGAGGTTACCCAG 3’  Reverse: 5’ ACCAGGGGAACCACTCTCA 3’ | NM_001113515.2 |
| mACAN | Forward: 5’ GTGGAGCCGTGTTTCCAAG 3’  Reverse: 5’AGATGCTGTTGACTCGAACCT 3’ | NM_007424.3 |
| mPRG4 | Forward: 5’ GATCCTGGGTATCCTAAA 3’  Reverse: 5’ TATAAGTGTACTGCTGAATG 3’ | NM_021400.3 |
| mCOL1A1 | Forward: 5’ GATAGGGACTTGTGTGAA 3’  Reverse: 5’ AGTGGAGAGAGAGTAGAG 3’ | NM_007742.4 |
| mCOLXA1 | Forward: 5’ TTCTGCTGCTAATGTTCTTGACC 3’  Reverse: 5’ GGGATGAAGTATTGTGTCTTGGG 3’ | NM_009925.4 |
| pRPL7 | Forward: 5’ CAGGATCAGAGGTATCAA 3’  Reverse: 5’ TATATGGTTCCACAATTCTC 3’ | NM_001113217.1 |
| pSOX9 | Forward: 5’ CCACCGAAGAAAGACCGTAA 3’  Reverse: 5’ CTTGGAATGTGGGTTCGAGT 3’ | NM_213843.2 |
| pACAN | Forward: 5’ CTACGACGCCATCTGCTACA 3’  Reverse: 5’ CTTCACCCTCGGTGATGTTT 3’ | NM_001164652.1 |
| pCOL2A1 | Forward: 5’ GAGAGGTCTTCCTGGCAAAG 3’  Reverse: 5’ AAGTCCCTGGAAGCCAGAT 3’ | XM_021092611.1 |
| pCOL1A1 | Forward: 5’ CCAGTCACCTGCGTACAGAA 3’  Reverse: 5’ ACGTCATCGCACAACACATT 3’ | LC223106.1 |
| pCOLXA1 | Forward: 5’ ACTTCTCCTACCACATTC 3’  Reverse: 5’ CCATACCTGGTCATTATCT 3’ | NM_001005153.1 |
| pPRG4 | Forward: 5’ TTGGCATCAACCCCATATTT 3’  Reverse: 5’ GTATCAATGGGGGAGGGAAT 3’ | XM_021063824.1 |
| pADAMTS5 | Forward: 5’ GAGGGGGCAACTCACATAAA 3’  Reverse: 5’ GGTTTCCGAAGTGGAGATCA 3’ | XM_021070865.1 |
| pMMP13 | Forward: 5’ TCTTGTTGCTGCCCATGAGT 3’  Reverse: 5’GTGGCTTTTGCCAGTGTAGG 3’ | XM_021062712.1 |
| hRPL7 | Forward: 5’ CCAAATTGGCGTTTGTCATCAG 3’  Reverse: 5’ GCATGTTAATCGAAGCCTTGTTG 3’ | NM_000971 |
| hCOL2A1 | Forward: 5’ TGGACGATCAGGCGAAACC 3’  Reverse: 5’ GCTGCGGATGCTCTCAATCT 3’ | NM_001844 |
| hSOX9 | Forward: 5’ AGCGAACGCACATCAAGAC 3’  Reverse: 5’ GCTGTAGTGTGGGAGGTTGAA 3’ | NM_000346 |
| hCOL1A1 | Forward: 5’ GAGGGCCAAGACGAAGACATC 3’  Reverse: 5’ CAGATCACGTCATCGCACAAC 3’ | LC223106.1 |
| hACAN | Forward: 5’ CTGCTTCCGAGGCATTTCAG 3’  Reverse: 5’ CTTGGGTCACGATCCACTCC 3’ | NM_001369268.1 |
| hPRG4 | Forward: 5’ AAAGTCAGCACATCTCCCAAG 3’  Reverse: 5’ GTGTCTCTTTAGCGGAAGTAGTC 3’ | NM_005807.6 |
| hCOLXA1 | Forward: 5’ CATAAAAGGCCCACTACCCAAC 3’  Reverse: 5’ ACCTTGCTCTCCTCTTACTGC 3’ | NM_000493.4 |

m = mouse, p = pig, h = human

**Table S2 - Antibodies used for IHC**

| **Antibody** | **Vendor** | **Catalog Number** | **Dilution** |
| --- | --- | --- | --- |
| Collagen II | Bioss | Bs-0709R | 1:200 – 1:500 |
| SOX9 | Millipore | AB5535 | 1:1000 |
| Collagen I | Santa Cruz Bio | Sc-59772 | 1:200 |
| Collagen X | Abcam | Ab49945 | 1:500-1:1000 |
| Aggrecan | Millipore | AB1031 | 1:200 – 1:500 |
| Anti-Mouse IgG ImmPRESS | Vector | MP-7422 | Pre-diluted |
| Anti-Rabbit IgG ImmPRESS | Vector | MP-7401 | Pre-diluted |
